# Can’t Intubate, Can’t Oxygenate Airway Failure Due to Post-thyroidectomy Cervical Hematoma: A Case Report

**DOI:** 10.7759/cureus.115648

**Published:** 2026-09-02

**Authors:** Michael Kostares, Georgios Noutsos, Efstratios Andritsos, Paul Stampouloglou, Maria Piagkou

**Affiliations:** 1 Anatomy and Surgical Anatomy, National and Kapodistrian University of Athens, Athens, GRC; 2 Otolaryngology - Head and Neck Surgery, Metaxa Hospital, Piraeus, GRC; 3 Anesthesiology, Metaxa Hospital, Piraeus, GRC

**Keywords:** airway obstruction, can’t intubate, can’t oxygenate, emergency cricothyrotomy, front-of-neck access, post-thyroidectomy cervical hematoma, thyroidectomy

## Abstract

Post-thyroidectomy cervical hematoma is an uncommon but potentially fatal complication that may rapidly progress to life-threatening airway obstruction. We report the case of a 49-year-old woman who developed severe respiratory distress, marked cyanosis, and loss of responsiveness approximately four hours after total thyroidectomy with central compartment neck dissection. Immediate bedside wound opening and hematoma evacuation were performed, but adequate oxygenation was not restored. Two attempts at tracheal intubation using direct laryngoscopy were unsuccessful because severe upper-airway edema and anatomical distortion prevented visualization of the glottis. Given the persistent failure of oxygenation and extreme urgency of the situation, emergency front-of-neck access was undertaken without further attempts at tracheal intubation. An emergency surgical tracheotomy was performed through the already opened thyroidectomy field, enabling effective ventilation. Subsequent surgical exploration identified active bleeding in the region of the previously ligated left superior thyroid artery, which was successfully controlled, and the emergency airway was converted to a formal tracheostomy. The patient recovered without residual airway-related complications and was discharged on postoperative day seven. This case emphasizes that bedside wound decompression may be insufficient once advanced upper-airway edema has developed and highlights the need for prompt escalation to emergency front-of-neck access when adequate oxygenation cannot be restored and tracheal intubation is unsuccessful.

## Introduction

Post-thyroidectomy cervical hematoma is an uncommon but potentially fatal complication because rapid bleeding within the confined cervical space may cause venous congestion, laryngopharyngeal edema, tracheal compression, and acute airway obstruction [1-3]. When airway compromise is suspected, immediate bedside wound opening and hematoma evacuation are recommended to decompress the neck [1]. However, decompression may fail to restore oxygenation when severe upper-airway edema and anatomical distortion have already developed. In this setting, tracheal intubation may be difficult or impossible, and persistent failure of oxygenation may necessitate emergency front-of-neck airway access, as recognized in established difficult-airway and post-thyroidectomy hematoma management guidelines [1,4,5]. We report a postoperative cervical hematoma after total thyroidectomy that progressed to life-threatening airway compromise despite prompt bedside decompression and unsuccessful tracheal intubation, ultimately requiring emergency front-of-neck access by surgical tracheotomy.

## Case presentation

A 49-year-old woman underwent total thyroidectomy with prophylactic central compartment neck dissection for a thyroid nodule with Bethesda category VI cytology on preoperative fine-needle aspiration. Her medical history included arterial hypertension and coronary artery disease. She was not receiving antiplatelet or anticoagulant therapy, and her preoperative platelet count and coagulation parameters were within normal limits. Preoperative airway assessment showed a Mallampati class I airway, with no known history of difficult airway. Tracheal intubation was uncomplicated and successful on the first attempt.

The procedure was completed without intraoperative complications and with minimal blood loss. The recurrent laryngeal nerves and parathyroid glands were preserved, hemostasis was confirmed before closure, and a closed-suction drain was placed. Extubation at the completion of surgery was uncomplicated, and the patient was transferred to the ward without airway-related concerns. No hypertensive episode was noted during the early postoperative period before the acute deterioration. Final histopathological examination confirmed papillary thyroid carcinoma.

Approximately four hours after surgery, the patient developed acute respiratory distress accompanied by rapidly expanding cervical swelling. On assessment by the emergency team, she was markedly cyanotic and unresponsive to verbal or painful stimuli. Given the immediately life-threatening presentation and need for urgent airway intervention, a complete set of vital signs, including pre-intervention oxygen saturation, could not be documented before treatment was initiated. A postoperative cervical hematoma causing acute airway compromise was clinically suspected.

The on-call otolaryngologist immediately reopened the cervical incision at the bedside, opening the superficial and deep layers of the thyroidectomy wound, including the strap muscles, and evacuated the hematoma using suction and gauze. Airway management proceeded simultaneously. The on-call anesthesiologist made two attempts at tracheal intubation using direct laryngoscopy; both were unsuccessful because severe upper-airway edema and anatomical distortion prevented visualization of the glottis. Given the patient’s marked cyanosis, persistent inability to restore adequate oxygenation despite cervical decompression, and the extreme urgency of the situation, face-mask ventilation, videolaryngoscopy, and supraglottic airway placement were not attempted. Following the second unsuccessful intubation attempt and persistent failure of oxygenation, immediate emergency front-of-neck access was undertaken.

The on-call otolaryngologist performed an emergency surgical tracheotomy through the already opened thyroidectomy field. With the trachea directly exposed, a transverse incision was made between the first and second tracheal rings, and a cuffed 8.0-mm internal-diameter endotracheal tube was inserted through the tracheal opening. Correct tube placement and effective ventilation were confirmed by direct visualization, bilateral chest expansion and auscultation, and capnography. The cuff was then inflated, and manual ventilation was initiated using a self-inflating bag. Endotracheal suctioning was subsequently performed to remove blood clots. Following establishment of the airway and effective ventilation, the patient’s cyanosis gradually improved, accompanied by progressive recovery of consciousness, initially with responsiveness to painful stimuli and subsequently with spontaneous eye opening. No immediate procedure-related complications were observed.

The patient was then transferred to the operating room for surgical exploration and definitive hemostasis. Active bleeding was identified in the region of the previously ligated left superior thyroid artery and was successfully controlled. The emergency tracheal airway was subsequently converted to a formal tracheostomy using a cuffed 8.0-mm tracheostomy tube.

The patient was admitted to the intensive care unit for postoperative airway monitoring. After 24 hours of clinical stability, she was transferred to the otolaryngology ward. Following clinical improvement of the upper-airway edema, flexible endoscopic airway assessment and a successful capping trial were performed, followed by successful decannulation on postoperative day five. The subsequent clinical course was uncomplicated, and the patient was discharged on postoperative day seven without residual airway-related complications. The clinical course from acute airway compromise through emergency airway management and subsequent recovery is summarized in Figure 1. Written informed consent was obtained from the patient for the publication of this case report and any accompanying clinical information.

**Figure 1:**
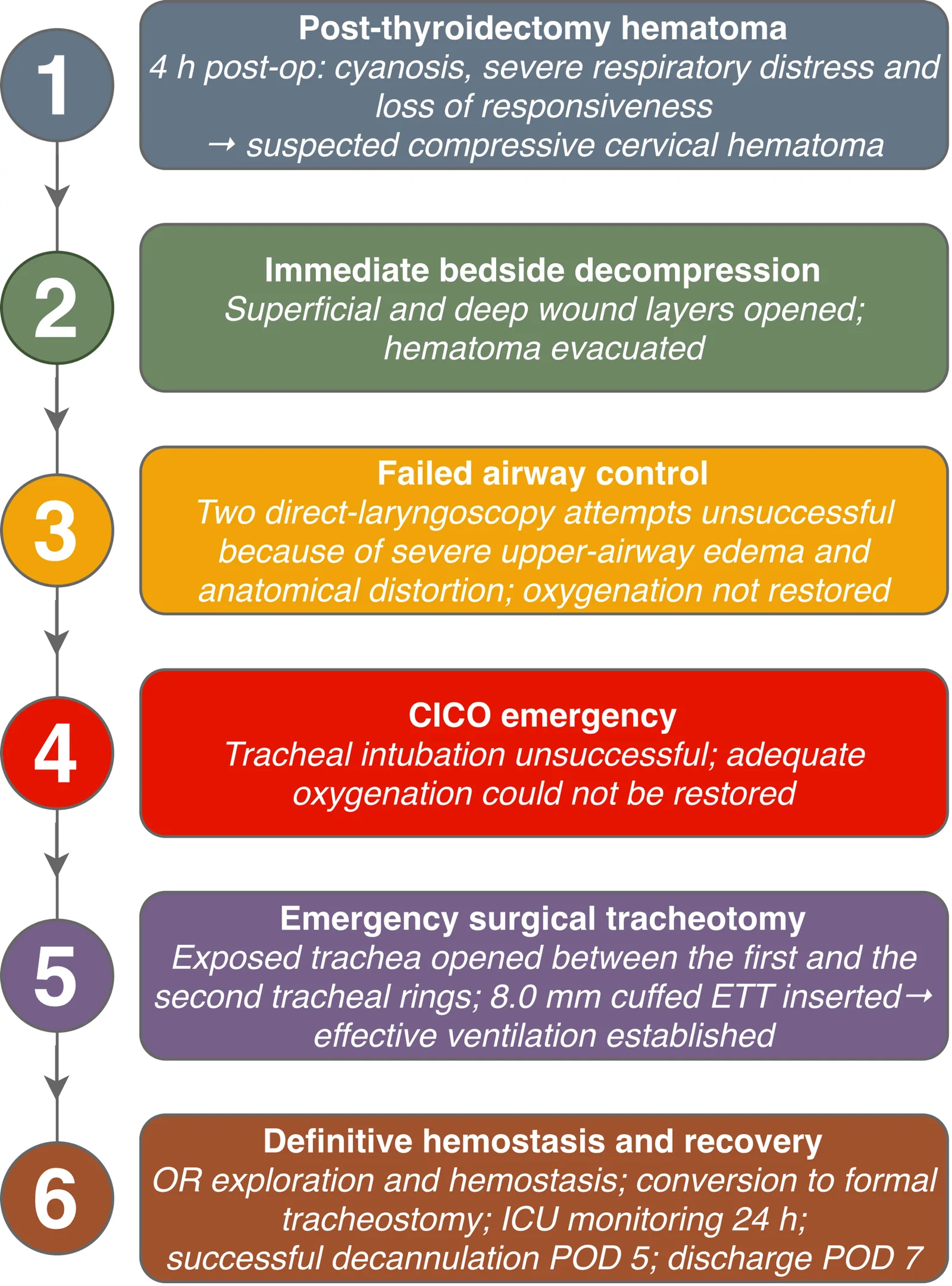
Clinical course and airway management of post-thyroidectomy cervical hematoma The figure summarizes the clinical course from recognition of acute airway compromise through immediate bedside wound decompression, unsuccessful tracheal intubation, persistent failure of adequate oxygenation, CICO emergency, emergency surgical tracheotomy, definitive hemostasis, conversion to formal tracheostomy, and subsequent recovery. CICO: can’t intubate, can’t oxygenate; ETT: endotracheal tube; OR: operating room; ICU: intensive care unit; POD: postoperative day

## Discussion

Post-thyroidectomy cervical hematoma is a time-critical complication in which airway compromise may develop rapidly through a combination of tracheal compression, impaired cervical venous drainage, progressive laryngopharyngeal edema, and distortion of normal airway anatomy [1,6]. The approximately four-hour interval to clinical deterioration in the present case is consistent with the recognized early postoperative timing of post-thyroidectomy hematoma. In a series of 3,086 thyroidectomies, 72.7% of hematomas requiring surgical re-exploration occurred within the first six postoperative hours [7], while a more recent analysis of 6,938 thyroid operations found that 95.8% of postoperative hemorrhages requiring reoperation occurred within the first 24 hours [8]. Recognition is primarily clinical, and neither diagnostic investigations nor transfer to the operating room should delay immediate bedside intervention when airway compromise is suspected [1].

Immediate opening of the cervical wound and evacuation of the hematoma are recommended as initial decompressive measures when airway compromise is suspected [1]. In the present case, immediate bedside decompression, including opening of the superficial and deep wound layers and evacuation of the hematoma, failed to restore adequate oxygenation in the setting of severe upper-airway edema and anatomical distortion. Notably, a postoperative cervical hematoma developed despite the presence of a closed-suction drain. Meta-analytic evidence indicates that routine drain placement after thyroidectomy is not associated with a significant reduction in postoperative bleeding or cervical hematoma [9]. The superior thyroid vessels are also a recognized source of postoperative bleeding. In a series of 5,900 patients undergoing thyroid surgery, an identifiable bleeding point was found in 58.1% of patients requiring reoperation for postoperative hematoma; superior thyroid vessels were the bleeding source in 12.9% of patients with postoperative hematoma [10].

Cervical decompression and airway management should proceed in parallel, with close coordination between the surgical and anesthetic teams. In the present case, two attempts at tracheal intubation using direct laryngoscopy were unsuccessful because severe upper-airway edema and anatomical distortion prevented visualization of the glottis. This difficulty is consistent with previous reports describing marked laryngeal or supraglottic edema and clinically significant airway compromise following post-thyroidectomy hematoma, which may substantially complicate airway management [6,11,12]. Current post-thyroidectomy hematoma guidelines recommend tracheal intubation when wound evacuation fails to resolve airway compromise and emphasize limiting repeated intubation attempts [1]. In the present case, face-mask ventilation, videolaryngoscopy, and supraglottic airway placement were not attempted because of the marked cyanosis, persistent inability to restore adequate oxygenation, and extreme urgency of the clinical situation. Following two unsuccessful direct-laryngoscopy attempts and failure of cervical decompression to restore adequate oxygenation, emergency front-of-neck access was undertaken without delay. Surgical front-of-neck access, including emergency tracheostomy, represents an airway rescue option in this setting [1,4,5]. Rapid escalation to emergency front-of-neck access has also been described in postoperative neck hematoma complicated by acute airway obstruction [13].

In the present case, emergency surgical tracheotomy through the already opened thyroidectomy field provided direct tracheal access and enabled effective ventilation, permitting subsequent transfer to the operating room for definitive hemostasis and conversion to a formal tracheostomy. Teams caring for patients after thyroid surgery should be prepared both to perform immediate bedside wound decompression and to proceed to emergency front-of-neck access when adequate oxygenation cannot be restored and tracheal intubation is unsuccessful. Clear role allocation, immediate availability of emergency airway equipment, and multidisciplinary simulation training may facilitate coordinated team responses and timely escalation during this uncommon but potentially fatal emergency [14].

## Conclusions

Post-thyroidectomy cervical hematoma may progress rapidly to life-threatening airway obstruction. In the present case, immediate bedside wound decompression did not restore adequate oxygenation in the setting of severe upper-airway edema and anatomical distortion. When adequate oxygenation cannot be restored and tracheal intubation is unsuccessful, repeated intubation attempts should be limited and emergency front-of-neck access should not be delayed. In this patient, persistent failure of oxygenation following cervical decompression and unsuccessful tracheal intubation prompted emergency front-of-neck access by surgical tracheotomy, which enabled effective ventilation and was followed by recovery without residual airway-related complications.
